# Critical success factors and challenges for individual digital study assistants in higher education: A mixed methods analysis

**DOI:** 10.1007/s10639-022-11394-w

**Published:** 2022-10-19

**Authors:** Claudia M. König, Christin Karrenbauer, Michael H. Breitner

**Affiliations:** Information Systems Institute, Leibniz University Hannover, Königsworther Platz 1, 30167 Hannover, Germany

**Keywords:** Individual digital study assistant, Critical success factors and challenges, IS success model, Technology in higher education, Mixed methods, Research agenda

## Abstract

During the COVID-19 pandemic, the availability of online higher education programs and tools has grown rapidly. One example is an individual digital study assistant (IDSA) for students, which provides functionalities to train self-regulation skills, to engage with own educational goals and to offer automated, first-level support to higher education institution (HEI) units and employees. An IDSA further can guide students through HEI and their administration. But, what are the critical success factors (CSF) and challenges for an IDSA? We deduce these using a mixed methods approach with one quantitative student survey, two rounds of interviews with various HEI experts, and a literature review. We classified our results according to the information system (IS) success model of DeLone & McLean (2016). Our results and findings show, e.g., that skilled and reliable HEI personnel, well-organized and useful content, cross-platform usability, ease of use, and students’ social factors are essential. Attractive IDSA functionalities are a major challenge because students use many apps, daily. Based on our CSF and challenges, we deduce theoretical and practical recommendations and develop a further research agenda.

## Introduction

Today’s students are confronted with a wide range of various degree programs, subjects, and courses. This is partly the result of various reforms that have taken place at higher education institutions (HEI), such as the Bologna Process in Europe or the Brandly Report in Australia. These reforms enable more students to study regardless of their social and educational background (Clarke et al., 2013; OECD, 2018; Van der Wende, 2000). Consequently, the number of students has increased and is more heterogeneous (OECD, 2018). In addition, individual needs and study goals are becoming more diverse. As a result, there is a growing need for personalized and individualized student advising and support (Wong & Li, 2019). In addition, students are now accustomed to receiving their information quickly and easily, also on mobile devices (Gikas & Grant, 2013). However, with student enrollment increasing and the number of lecturers and student support employees remaining almost unvaried (Hornsby & Osman, 2014), human support and advising alone is hardly feasible (Marczok, 2016). The current COVID-19/22 pandemic has further exacerbated this situation and changed pre-pandemic routines at HEI. Since then, studies have been dominated by online lectures with few face-to-face courses. As a result, face-to-face advising and mentoring are less possible, which increases the importance of self-organization and goal-oriented learning (i.e., students’ self-regulatory skills). However, according to Traus et al. (2020), students often have intrinsic motivational difficulties, and self-organization topics are perceived as difficult because of perceived extra work and the uncertainty of overlooking essential topics.

Through digital transformation (DT), digital assistants emerged and increased in importance in the educational context (Fitzgerald et al., 2013). They enable to meet the various changes and challenges in the HEI context and allow digital student support along face-to-face advising and counseling (Abad-Segura et al., 2020). Much research has already been conducted on pedagogical conversational agents (PCA; Wollny et al., 2021) that support students in learning (e.g., Hobert 2019; Ruan et al., 2019). In addition, individual digital study assistants (IDSA) offer the opportunity to strengthen self-organization and self-regulation skills and enable individualized support through personalized recommendations and reminders to address the trend of individualization and increasing student numbers. IDSA can incentivize students to be more active in pursuing their own educational goals, providing attractive features to help them, and thus practice self-regulated studying. They further offer first-level support for organizational units and thus relieve the work of advisers (blinded for review). However, there must be a fundamental readiness to accept this kind of support and grapple with it (Keramati et al., 2011).

In addition, to increase the likelihood of a successful IDSA implementation and usage and to address the challenges in the HEI context, it is crucial to have a more detailed understanding of the influencing factors. Therefore, we identified critical success factors (CSF) and challenges for IDSA implementation and usage. For this purpose, we applied a mixed methods research design (Creswell et al., 2003) with a quantitative student survey (n = 570), qualitative expert interviews (n = 28) and conducted a literature review parallel to the empirical part of our work. These various perspectives were crucial to determine the current state of research and practice and to learn from stakeholders in the field what an IDSA for HEI requires to be potentially used. Our results and findings contribute to the knowledge base of digital assistants in the HEI context; further, they are beneficial for the IDSA design, development, and implementation process and can be used by HEI for that. They can also support the selection process of an IDSA for HEI and enable existing systems to be further developed. In this respect, we concentrate on the following research question (RQ):

- What are critical success factors and challenges for an individual digital study assistant in higher education?

To answer our RQ, we review the theoretical foundations of self-regulation, IDSA in HEI, CSF for IDSA and an IS success model. We then describe our mixed methods research design using a quantitative survey, qualitative interviews, and a literature review to extract CSF and challenges for IDSA. They are subsumed into dimensions of the DeLone and McLean’s IS success model (DeLone & McLean, 2016). We discuss our results and findings, deduce implications and recommendations for research and HEI and propose a research agenda. We conclude with limitations and conclusions.

## Theoretical background

### Self-regulation

Wolters and Hussain (2015) attribute self-regulatory skills for self-study as having a major impact on successful HEI graduation. In addition to study skills, self-regulated learning includes data literacy (Janson et al., 2021), becoming increasingly important in the context of more individualized studies. Bandura (1986) emphasizes this as the ability to consciously set goals and monitor the extent to which these goals are achieved. Carver and Scheier (2011) and Zimmerman (2012) tie into the goal-oriented aspect of Bandura’s (1986) understanding of self-regulated tasks as a cyclical process. Influences and changes from the pervasive DT offer opportunities to address these challenges (Legner et al., 2017). The differentiated skills that self-regulated work requires (such as on the micro-level [e.g., reception of a particular text], the meso level [e.g., time management within a course], and the macro level [e.g., general organization of studies]) are summarized under the term self-observation (Vanslambrouk et al., 2018). This refers to the observation of one’s current goal-related behavior, which allows to determine whether the strategies used serve to achieve the goal in the sense of a target-actual comparison (Schunk, 2005). For goal-oriented self-observation in the context of a study, extrinsic factors must be considered in addition to internal processes such as intrinsic motivation and attention (Heckhausen & Heckhausen, 2018; Pintrich, 2000). Extrinsic factors include study-related information and resources.

### IDSA in HEI

The DT in HEI is characterized by dynamics, digitization processes, knowledge and skills transfer changes, new teaching and learning opportunities, changing organizational eco-systems, requirements, and legal frameworks (Bond et al., 2018). Digital assistants provide solutions to support individuals and organizations in the dynamic conditions and demands arising from DT (Murphy, 2020). Various HEI, for instance, use virtual assistants, also known as chatbots, to supplement existing offers (Bouaiachi et al., 2014; Hobert, 2019; Ranoliya et al., 2017). According to Knote et al. (2019), chatbots are one of five archetypes of smart personal assistants (SPA). Chatbots applied specifically in the educational context are called PCA (Wellhammer et al., 2020). They combine a natural language interface and artificial intelligence with a knowledge base. This intelligent human–computer conversation allows giving answers, hints, and suggestions for the user’s questions (Meyer von Wolff et al., 2020; Mikic et al., 2009; Winkler & Söllner, 2018). Researchers and practitioners have already introduced many different assistants, which, for example, support students to learn to write program code, strengthen their argumentation skills, answer FAQs, or support study course selections (Bouaiachi et al., 2014; Hobert, 2019; Ranoliya et al., 2017; Wambsganss et al., 2020).

Additional digital assistants resulting from the DT are IDSA. They can be categorized into one of the five archetypes identified by Knote et al. (2019), dependent on their design, architecture, and functionalities. IDSA enable first-level support for students. Their situation-specific and individualized recommendations, reminders, and advice enable students to plan and manage their studies more efficiently. The primary goal of an IDSA is to improve self-regulation skills, goal achievement, and study organization, providing appropriate functionalities (blinded for review). An IDSA deals with learning content on a reflective level through its functionalities. As opposed to PCA, IDSA support individual study structuring and situation-specific recommendations rather than being a content learning support. Therefore, they provide functionalities such as, for example, major and study course selection based on a self-assessment, individual learning strategy recommendations, and open educational resources (OER) and teaching networks suggestions (blinded for review process). Using an IDSA can compensate for the increasing heterogeneity and related individual needs. To design and implement an IDSA successfully, it is essential to consider essential key factors. Therefore, we systematically deduce CSF and challenges for IDSA in the following sections.

### Critical success factors and IS success model

CSF have been a much-researched topic in ISs since many years (Lee & Ahn, 2008; Hawking & Sellitto, 2010; Daniel, 1961) was one of the first to introduce this topic. Accordingly, to avoid information overload, companies must focus on a limited number of key factors. Rockart (1979) and Bullen and Rockart (1981) further expanded and built on it. They define CSF as “[…] the limited number of areas in which satisfactory results will ensure successful competitive performance for the individual, department or organization. CSF are the few key areas where “things must go right” for the business to flourish and for the manager’s goals to be attained” (Bullen & Rockart, 1981, p.7; Rockart 1979, p.84–85). Similarly, Leidecker and Bruno (1984) define CSF as “those characteristics, conditions or variables that when properly sustained, maintained, or managed can have a significant impact on the success of a firm competing in particular industry” (p. 24). In general, much research has already been conducted on CSF in various research topics: for IT projects in general (e.g., Trigo & Varajão 2020), enterprise resource planning implementation projects (e.g., Sousa 2004), business process management (e.g., Trkman 2010), and also in the educational context (e.g., Alhabeeb & Rowley 2018).

**Fig. 1:**
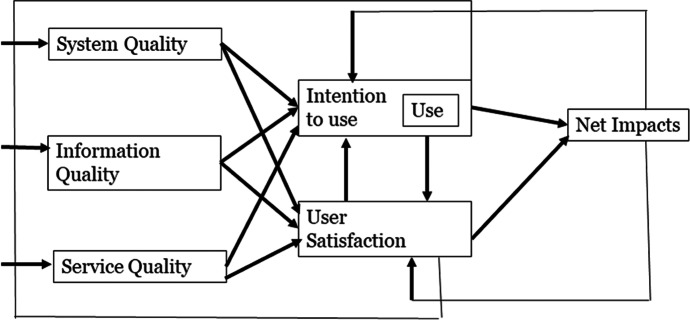
Updated DeLone and McLean IS success model (DeLone & McLean, 2016)

In addition to CSF, further methods and theories in research explain usage behaviors and technology success. One example is the IS success model by DeLone and McLean (2016), which we used for the identified CSF and challenges and assigned them to the dimensions, see Fig. 1.

Delone and McLean (2016) reviewed the numerous publications published in 1981–1987 to develop a taxonomy of IS success, in other words, which factors are critical to IS success. This taxonomy was based on Mason’s (1978) modification of Shannon and Weaver’s (1949) communication model, which identified three levels of information: the technical level, which refers to the accuracy and efficiency of the system producing the information; the semantic level, namely the ability to convey the intended message; and the effectiveness level, meaning the effect on the receiver. Mason adapted this theory for the IS community and expanded the effectiveness level into three categories: receipt of information, impact on the recipient, and impact on the system (Mason, 1978).

The working group around Masson identified categories for system success assigning one aspect of IS success to each of Mason’s (1978) effectiveness levels. From this analysis, six variables for IS success emerged: system quality, information quality, usage, user satisfaction, individual impact, and organizational impact, the last two of which were obtained after other researchers modified the model for their research, that is, the initial model, 10 years later, in 2003. System quality corresponded to the technical level of communication, while information quality corresponded to the semantic level of communication. The other four variables corresponded to Mason’s (1978) subcategories of the effectiveness level. Usage was related to Mason’s (1978) receipt of information, and user satisfaction and individual impact were related to the impact of information on the recipient, while organizational impact was the impact of information on the system.

The IS success model is well established in the IS community and provides us with an opportunity to classify CSF into established categories. The model shows the interdependence of the individual dimensions. To satisfy the potential user so that the IS offers motivations for it to be used, a number of well-functioning qualities are required: system quality, information quality, service quality, and the net impact measured by these.

*System Quality* is defined as “desirable characteristics of an IS” (Petter et al., 2014, p. 11). Improving IS, the variability in the system quality dimension decreases. Thus, user expectations can be better met, and this dimension has a lower influence on outcomes. Nevertheless, according to DeLone and McLean (2016), it remains important for IS success.

*Information Quality* is defined by the authors as “desirable characteristics of the system output, such as content, wording, reports, and dashboard” (Petter et al., 2014, p. 11). According to DeLone and McLean (2016), the information quality dimension is often not included in IS success analyses, even though it is an important dimension as it ensures accurate, timely, and relevant information.

*Service Quality* is by definition a “quality of the service or support that system users receive from the IS organization and IT support personnel in general or for specific IS” (Petter et al., 2014, p. 11). The service quality dimension is the most wrongly understood and neglected dimension within the IS success model. Thereby, together with the information quality dimension, neglect can negatively influence successful outcomes and lead to confusing results (DeLone & McLean, 2016).

*User Satisfaction* is defined as “users’ level of satisfaction with the IS” (Petter et al., 2014, p. 11). User satisfaction is a result of system success. According to DeLone and McLean (2016), it is important to measure this dimension holistically to capture system satisfaction.

*Net Impact* is defined as “extent to which IS are contributing to the success of individuals, groups, organizations, industries, and nations” (Petter et al., 2014, p. 11). It is a further dimension of IS success by DeLone and McLean (2016) and explains it as the most dependent and multifaceted success dimension as this construct measures the target outcome. The net effect is focused on the target and cannot provide significant results and findings due to numerous (human) influencing factors.

## Research design and methods

To identify and determine CSF, qualitative and quantitative approaches are often used with various methods, all of which have advantages and disadvantages (Sousa, 2004). To address the advantages of each method, many CSF are therefore collected based on mixed methods research (Tashakkori & Teddlie, 2003). Thus, we combined qualitative and quantitative methods along with a literature review (Flick, 2017, 2018; Johnson et al., 2007), to first identify and understand the needs of students, needs of HEI organizational units, needs of lecturers, and the current literature for a customized digital study assistant. We used the convergent parallel design (Creswell & Planko Clark, 2018; Kerrigan 2014) for a multi-perspective view of potential stakeholders such as students, HEI organizational units, and lecturers (quantitative-qualitative-qualitative approach). With parallel research, the results of one research strand were excluded from the other for their methodological aspects and did not affect the successive explorations with HEI stakeholders. Since our studies were conducted relatively independently, the mixing of the study results occurred largely in the data interpretation phase. We were motivated by the idea and common focus of our research to develop an IDSA and capture as many perspectives as possible, then analyzing the results under the question of what factors are important for stakeholders to use the study assistant. The research methods used are justified and described below. Table 1 provides an overview of our research design and the research methods used. In the end, we triangulated our results. It serves as a meta-view from different perspectives (Creswell & Plano Clark, 2018; Flick, 2017; Flick et al., 2012). Through this review, we had the opportunity to re-analyze our data to answer our RQ and explore the CSF and challenges for IDSA in HEI.

Finally, we used the triangulation method developed by Denzin (2009) and empirically studied by Flick (1992, 2017, 2018). In which the study of the same research subject of using different methods emphasizes the aspect of improving validity by identifying congruent results to summarize in a meta-view the results and findings. The answers to the research question are provided from different perspectives (Creswell & Plano Clark, 2018; Flick, 2017; Flick et al., 2012). Through this review, we had the opportunity to reanalyze the data to answer our RQ and identify CSFs and challenges for IDSA in HEI.

**Table 1:** Research design and methods

	Mixed methods research (convergent)		
	Step 1: Quantitative Analysis	Step 2: Qualitative Analysis	Parallel: Literature Review
Tasks	1. Questionnaire development 2. Pretest & adaptions 3. Study execution 4. Data analysis	1. Interview guideline development 2. Pretest & adaptions 3. Conducting interviews & transcribing 4. Data analysis	1. Keyword-based literature research 2. Definition of inclusion and exclusion criteria 3. Forward-, backward-, author, and similarity search 4. Systematic literature analysis
Method & references	Qualitative and quantitative data analysis (Corbin & Strauss, 2014)	Semi-structured and guideline-oriented expert interviews with inductive analysis (Corbin & Strauss, 2014; Johnston & Warkentin, 2010)	Literature review following PRISMA (Moher et al., 2009)
Data	570 students from three HEI with various studies	19 experts from various organizational units & 9 lecturers	Springer Link, IEEE Xplore, Wiley, Sagepub, Science Direct, Jstor, Taylor and Francis, AISeL, ACM Digital Library, Google Scholar
Results & findings	CSF and challenges from the student perspective	CSF and challenges from the perspective of organizational HEI stakeholders and lecturers	State of the art of current literature to extract CSF and challenges

### Quantitative analysis

First, we conducted an online survey with students from three German HEI and distributed the survey through the local learning management systems. Participation was completely voluntary, and all participants agreed to use their anonymized data for research purposes. To identify first CSF and challenges, the questionnaire consisted of five questions (cf. Appendix 1). Before conducting the survey, we performed a pretest with professors, research staff, and student assistants to determine whether all questions were easily understandable and efficient for answering our research question. A pretest is an initial testing of some or all instruments to ensure no unexpected difficulties during the study (Boudreau et al., 2001). Based on the feedback, we adapted our questions and made them gender neutral, thus finalizing the questionnaire. The first question was about the student’s sociodemographic data, while the second one was about important and useful functionalities for an IDSA and allowed multiple selections. The students here selected between 18 items; for instance, the study assistant provides the exam experiences of fellow students or information about learning materials and resources freely through openly licensed OER. In the third question, participants had to prioritize characteristics required for an IDSA on a 10-point Likert scale ranging from 1 “unimportant” to 10 “very important” (Roberts et al., 1999). These included selection possibilities, such as easy usability or factual orientation without gamification. All the items were ordered in a randomized order. Questions four and five allowed text entries and addressed still unnamed important aspects for an IDSA and the barriers to use it. As the study was conducted in German HEI, the questions were all in German. On average, it took 5 min to answer them all. We used a spreadsheet program to analyze the quantitative data for the first three questions. Afterward, the first two authors independently categorized the qualitative data according to Corbin and Strauss (2014) for the last two questions. Quotations within this paper were translated into English using committee-based parallel translation (Douglas & Craig, 2007). In total, 570 students from the three HEI participated in our survey. Of these students, 58% were female, 39% were male, 92% were aged between 18 and 29 years, 68% were bachelor’s students (primarily in their first four semesters), and 28% were master’s students. To allow a cross-sectional analysis, we acquired students from various studies, e.g., management and economics, humanities, computer science, engineering, law, and teacher education.

### Qualitative analysis

Second, we performed semi-structured and guideline-oriented expert interviews with employees from various organizational units (INT.U.) and lecturers (INT.L.) from various departments of one German HEI (Table 2). The potential participants (n = 49; 19 HEI employees and 30 lecturers) were contacted via email and, upon agreement to participate, were interviewed in person by the first author. Again, participation was completely voluntary, and all interviewees agreed to the use of their anonymized interview data for research purposes. Our interview sample intended to obtain the overall opinions and impressions about CSF and challenges for IDSA in HEI. All interviewees had broad HEI experience and direct or indirect contact with students and know typical problems, issues, and support potentials and therefore can contribute to answering our research question. Prior to the interviews, we performed a pretest to ensure that the interviewees would have no difficulties comprehending our interview guideline (Johnston & Warkentin, 2010). Therefore, we distributed the interview guideline among several professors, scientists, and student assistants and asked for its comprehensibility and efficiency. As it was already clear and comprehensible for all participants, no adaptions were necessary. The finalized interview guideline with open questions in shown in Appendix 2. We started the interviews with an introduction of ourselves and then asked about the expectations, requirements, CSF, challenges, and organizational eco-systems for successful IDSA implementation, operation, and usage (cf. Appendix 2). In total, we interviewed 19 experts from various organizational units and nine lecturers, until we reached a theoretical saturation (Corbin & Strauss, 2014) and stopped performing more.

**Table 2:** Organizational units / institutes of interview partners

Interview	University Organization/Institution	Interview	University Organization/ Institution
INT.U.1	Academic Examination Office	INT.U.15	Registration Office
INT.U.2	Central Study Guidance Center	INT.U.16	Campus Sport Center
INT.U.3	Service Center Teaching Evaluation and Process Support	INT.U.17	University Library
INT.U.4	Deans’ Office: Coordination of Degree Program	INT.U.18	School Office
INT.U.5	Deans’ Office	INT.U.19	International Office of a School
INT.U.6	Language Center		
INT.U.7	University Top Management	INT.L.1	Institute for Environmental Economics and World Trade
INT.U.8	Psychological-Therapeutic Support Center	INT.L.2	Institute for Sociology
INT.U.9	Study Finance Center	INT.L.3	Institute of Vocational Education and Adult Education
INT.U.10	Social Counselling Center	INT.L.4	Information Systems Institute
INT.U.11	E(lectronic)-learning Service Center	INT.L.5	House of Insurance
INT.U.12	International Office of the University	INT.L.6	Institute of Philosophy and History of the Natural Sciences
INT.U.13	Ombudsman for Studies and Teaching	INT.L.7	Institute of Applied Mathematics
INT.U.14	Central Institution for quality Development in Studies and Teaching	INT.L.8 INT.L.9	Institute of Criminal Science English seminar for students in teacher education

All interviews lasted 30–60 min. They were performed in German, recorded, transcribed, and analyzed qualitatively following Corbin and Strauss (2014) with MAXQDA 18 support. In general, the coding process is a procedure that “gets the analyst off the empirical level by fracturing the data, then conceptually grouping it into codes that then become the theory which explains what is happening with the data” (Glaser, 1978, p.55). The first two authors independently performed the coding process and then compared and discussed their codes until an agreement was reached on the final categories. First, we used open coding, a line-by-line coding (Wiesche et al., 2017), to identify initial patterns or labels within our data. After we labeled all contents of the transcript, we used selective coding to understand the relationships between the first identified labels and to identify more patterns (CSF, challenges). Afterward, we applied selective coding to determine the content related to and that specifies the previously identified patterns. Thereby, the coding process was iterative, with backward and forward movements to refine categories. Aligning with van Nes et al. (2010), we performed the coding and inductive analysis in German, preserving the original language as long as possible to avoid translation errors and limitations. Quotations for this paper were translated into English to support and substantiate our statements. We used committee-based parallel translation (Douglas & Craig, 2007) to ensure accuracy and maintain the meaning of the statements through the translation process. Therefore, the first two authors translated the statements from German to English separately, compared and discussed their results together with the third author, and decided for the best suitable translation or made adaptions (Douglas & Craig, 2007; McGorry, 2000).

### Literature review

**Fig. 2:**
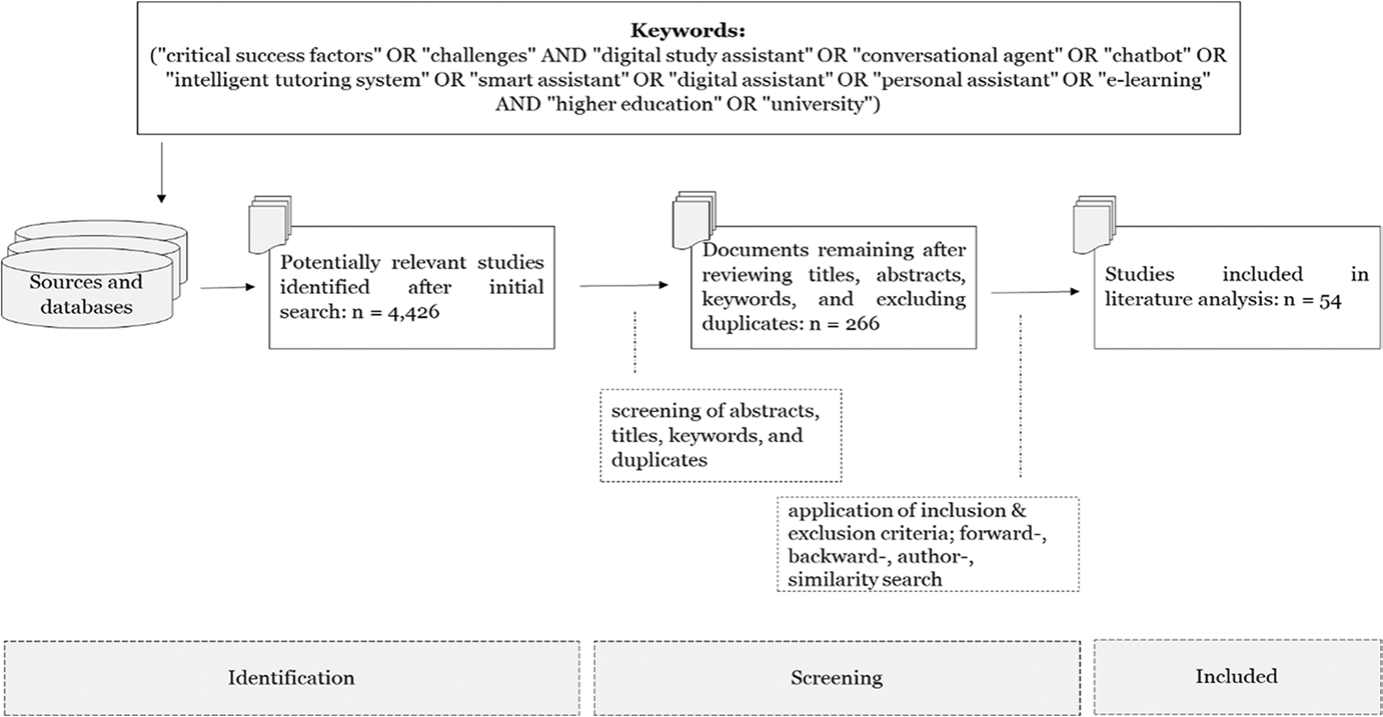
Literature review process (Moher et al., 2009)

At the same time, we performed a literature review following the principles of Preferred Reporting Items for Systematic Reviews and Meta-Analyses (PRISMA) to identify common CSF and challenges for IDSA (Moher et al., 2009). First, we searched for relevant articles in scientific databases, namely Springer Link, IEEE Xplore, Wiley, Sagepub, Science Direct, Jstor, Taylor and Francis, AISeL, ACM Digital Library, and Google Scholar. We here used the search string (“critical success factors” OR “challenges” AND “digital study assistant” OR “conversational agent” OR “chatbot” OR “intelligent tutoring system” OR “smart assistant” OR “digital assistant” OR “personal assistant” OR “e-learning” AND “higher education” OR “university”). Our initial literature search resulted in 4,426 scientific papers. We excluded duplicates, reviewed titles, abstracts, and keywords as well as papers not suitable to answer our research question. This led to 4,160 exclusions, with 266 papers remaining. We then defined inclusion and exclusion criteria to ensure a quality standard and reduce selection biases. Articles were included if they (1) were written in English or German, (2) were peer-reviewed in a journal or conference, (3) named or described CSF or challenges for IDSA, and (4) focused on an HEI context. Even though our IDSA is not a learning assistant, we also included (5) CSF and challenges for e-learning assistants that apply to an IDSA. The exclusion criteria were the following: (1) non-scientific articles, (2) abstract-only articles, (3) articles not accessible through HEI services or memberships, and (4) articles that named or described no CSF or challenges for IDSA. We analyzed the remaining 266 articles in more detail and applied the inclusion and exclusion criteria to determine the final sample, for which we conducted a forward-, backward-, author, and similarity search (Google Scholar). Finally, this manual selection resulted in 54 scientific papers for our literature review (Fig. 2).

## Results and findings

We used the IS success model by DeLone and McLean (2016) to structure the identified CSF and challenges for IDSA in HEI into the six IS success dimensions (cf. Figure 1). In the following, we describe our results and findings in more detail, and Table 3 gives a comprehensive overview of them.

### System maturity and quality

A first CSF and challenge for IDSA within the system maturity and quality dimension refers to the ease of use. According to Freeman and Urbaczewski (2019), inter alia, it includes an intuitive, user-friendly interface, which Al-Sharhan et al. (2010), Lu and Dzikria (2019), and Naveh et al. (2010) concretize with easy organized navigation and usage. According to our survey results, 53.84% of the students rated an IDSA’s easy usability and interface as one of the most important characteristics. Further, the lack of easy and intuitive usage is one of the biggest challenges of using an IDSA (student survey). For example, one expert added: “I see barriers in the usability quite clearly, that must be self-explanatory. If I have to study a manual, no one will use it” (INT.U.18). The second CSF and challenge contribute to an IDSA’s easy access. Within our student survey, participants mentioned the time-consuming registry and registration process as one challenge of an IDSA usage, which is also supported by the literature (Alhabeeb & Rowley, 2018; Freeman & Urbaczewski, 2019). Further, an IDSA’s flexibility represents an additional CSF and challenge. According to Raspopovic and Jankulovic (2014), a flexible adaption and personalization of an IDSA contributes to the system’s maturity and quality. Our survey results show that students want a modular design and the possibility to individualize an IDSA; it must not “[be] overloaded with unnecessary functions. Or at least the possibility of not having to use them” (student survey). Our survey further reveals the possibility of using an IDSA offline as another critical component for students.

IT maturity is another CSF and challenge within the system maturity and quality dimension. Here La Rotta et al. (2020) and Mosakhani and Jamporazmey (2010) especially see the system’s reliability, accessibility, guidance, timeliness, and the technologies’ actuality as fundamental. Our survey results further show that a test phase for error identification contributes to the IT maturity dimension. One student stated that “an extensive testing phase before [an IDSA] is made available to all students to avoid as many errors as possible later on,” and one expert added that “the important thing is that it [an IDSA] works, so the technology is important. If it doesn’t work once and a second time, the whole thing is off the table” (INT.U.17). Data privacy and security is another identified CSF and challenge within the system maturity and quality dimension. This includes protecting personal data, transparent handling, and anonymous data collection. An IDSA must further provide the possibility of data settings and deletion and prevent the misuse of personal data. One student, for instance, stated that they wanted “complete transparency in handling personal data and the option to reject individual aspects of use if necessary.” Several interviewees also stated the importance of data privacy and security: “So […] of course data protection is very, very important […]. I think it’s also very important to tell everyone immediately […] that they don’t have to be concerned that their data will be sold” (INT.U.10); “Before students agree to the use of their personal data, if applicable, it must be explained to them what they get in return (benefits) and why [an IDSA] then makes better recommendations” (INT.U.15). In addition, Alsabawy et al. (2011) state that stable security and adequate data transmission and communication are essential to achieve user trust.

### Information quality

Holsapple and Lee-Post (2006) and Raspopovic and Jankulovic (2014) state that content must be well organized, consistent, clearly written, systematic, useful, customizable to the individual needs, relevant, and up to date. Among others, Mosakhani and Jamporazmey (2010) and Naveh et al. (2010) emphasize that content must be sufficiently available and understandable to reach a high-quality standard. Further, students do not want redundant information and recommendations, nor information overload. They also emphasize that unreliable and outdated information are a challenge to use an IDSA. In addition, another identified CSF is data integration. An IDSA must allow the portability of previous data to counteract the usage challenge of manually entering many data, which also allows to link existing data and make new recommendations based on them; “it [an IDSA] gives a good overview of the enormous amount of data that a study brings with it and based on that makes optimized suggestions. For example, evaluating one’s own grades from previous exams to be able to name further courses based on this” (student survey).

### Service quality

Bani-Salameh and Abu Fakher (2015), La Rotta et al. (2020), and McPherson and Nunes (2006) highlight the importance of skilled personnel to enable professional and efficient technical support and maintenance. According to Soong et al. (2001), this includes, in addition instructor training, answering ongoing questions during the semester from both students and instructors. Therefore, students want to “contact persons for problems and suggestions for improvement” (student survey), and an IDSA “refers me to the right contact persons for possible questions” (student survey). Fabito (2017) suggests a “holistic support provided by the management to support the implementation (…)” (p. 222). Other CSF and challenges are answer quality and employee responsiveness. Answers must be fair and knowledgeable so that students and faculty can rely on them (Holsapple & Lee-Post, 2006). In addition, employees must be able to respond to requests in a timely manner (La Rotta et al., 2020; Naveh et al., 2010).

### User satisfaction

Holsapple and Lee-Post (2006) state that positive experiences, recommendation to others, and involvement in the design process (McPherson & Nunes, 2006) contribute significantly to user satisfaction. According to Odunaike et al. (2013), the sustainability and up-to-dateness of information and content development and maintenance are also critical factors in increasing user satisfaction. Especially for students, platform independence or cross-platform usability is a further CSF and challenge for an IDSA. A frequently mentioned aspect is system independence, so that an IDSA is “available and compatible on all operating systems, browsers, and smartphones, also as an app” (student survey). In addition, portals and platforms used by HEI must be integrated into an IDSA. “Linking possibilities with already existing online platforms […] is very important, otherwise redundancies and overlaps arise” (student survey). Experts also highlight the importance of this CSF to ensure that students are not forced to use different systems.

### Net impact

Raspopovic and Jankulovic (2014) emphasize the importance of learning enhancement, academic achievement, time savings, or knowledge gain. Holsapple and Lee-Post (2006) point out that net impact has positive and negative effects. Positive effects are, for instance, learning enhancement, empowerment, time savings, and academic achievement, while negative effects include lack of content, isolation, quality concerns, and technology dependency. The experts in our interviews are convinced that the net impact increases when the IDSA has reached an HEI’s top management; then, it can be more purposefully communicated to students and financially supported: “it has to get into the heads, that is, the heads of the university management and the entire higher education institutions, if you want to be successful with it. Otherwise, as we’ve seen many times before, it’s not going to work” (INT.U.14). Students see the net benefit in the added value that the IDSA must bring in terms of exciting functionalities, and lecturers see the net impact on the credibility of relevant content and recommendations: “if the assistant conveys credibility, that is, if this is a credible tool where you really have the feeling that these are meaningful recommendations that don’t just come from somewhere, but are also somehow well-founded, then I could imagine that this could also be a relief for students” (INT.L.6).

### Intention to use

Fabito (2017), Bani-Salameh and Abu Fakher (2015), Mosakhani and Jamporazmey (2010), and Selim (2007) state that motivation is the most critical aspect for users to remain active. Hao et al. (2017) highlight perceived usefulness. For Odunaike et al. (2013), the fundamental willingness to be open to use is critical. Tarhini et al. (2013) see social factors as crucial determinants of use and the role of peers and lecturers influencing use as another success factor. Furthermore, self-regulation/organization (Eom & Ashill, 2016; Miranda et al., 2014)—in other words, the extent to which a student is able to act in a self-regulatory and well-organized manner—is a factor; “it’s a good idea to encourage self-regulation and especially to set goals, on the other hand, students want to know exactly what exam content is required” (INT.L.1). Different languages show circumspection and convenience for the user (Lu & Dzikria, 2019). Experts see addressing defined target groups as an opportunity to address student groups in a subject-specific way—for example, international students from one country, rather than complete, non-specific individualization for all students: “what I could imagine here is to take the international students more by the hand because they don’t know the clientele. So they get to know our system better and get in touch with others, otherwise, they stay in their community” (INT.U.13). The faculty sees the benefit in individualized offers; “if you do make individualized offers to students, and say, we’ve seen you’re interested in this […] that is, perhaps for us to know better what the student wants […] a great added value” (INT.L.4).

The results of our qualitative and quantitative studies allowed to triangulate our findings (Flick, 2018), meaning that the data are based on various sources and allow various perspectives on our defined goal. We chose the “in-between method,” which allows for a methodological mix of the chosen survey instruments (Flick, 2018). We examined the question of what CSF and challenges influence IDSA from a bird’s eye view. The experts from the organizational units agree that the following factors are important for the success of an IDSA: one platform for everything, useful functionalities for students so that they recognize added value, intuitive operation, and comprehensive information on data protection. The lecturers need to promote self-determined learning and the willingness to make decisions. Those who work with incoming students generally say that the incoming students must be taken more by the hand in order for them to get to know the HEI system more quickly and that ease of use and data protection must play a more significant role in the sense that the students must be more concerned with which of their data they release for which purpose. Students see the following as CSF and challenges: attractive functionalities, realistic recommendations, one platform, and (ideally) an all-in-one solution. We subsumed these into the dimensions of the model of DeLone and McLean (2016; see Table 3).

**Table 3:** Final results: CSF and challenges for IDSA in HEI

	IDSA CSF and challenges	References
System Maturity and Quality	Ease of use (intuitive, user-friendly, easy organized navigation and usage, usability and interface, self-explanatory)	Al-Sharhan et al. (2010); Freeman and Urbaczewski (2019); Lu and Dzikria (2019); Naveh et al. (2010); expert interviews; student survey
	Easy access (no time-consuming registry process, easy registration and access)	Alhabeeb and Rowley (2018); Freeman and Urbaczewski (2019); student survey
	Flexibility (offline usage, flexible adaption and personalization, modular design, individualization)	Raspopovic and Jankulovic (2014); student survey
	IT maturity (system’s reliability, accessibility, guidance, timeliness, actuality)	La Rotta et al. (2020); Mosakhani and Jamporazmey (2010)
	Test phase for error identification	expert interviews; student survey
	Data privacy and security (personal data protection, transparent handling, anonymous data collection, data settings, data deletion, prevent misuse of personal data)	Alsabawy et al. (2011); Lu and Dzikria (2019); expert interviews; student survey
Information Quality	Content (well organized, consistent, clearly written, systematic, useful, customizable to the individual needs, relevant, up to date, sufficiently available, understandable to reach a high-quality standard)	Al-Sharhan et al. (2010); Holsapple and Lee-Post (2006), Mosakhani and Jamporazmey (2010); Naveh et al. (2010); Raspopovic and Jankulovic (2014)
	No redundant information, no information overload, reliable information	Student survey
	Data integration (portability of previous data, link existing data)	Student survey
Service Quality	Skilled personnel (technical support and maintenance, instructor training, answering of ongoing questions, contact persons)	Bani-Salameh and Abu Fakher (2015); Fabito (2017); La Rotta et al. (2020); McPherson and Nunes (2006); Soong et al. (2001); student survey
	Answer quality and employee responsiveness (fair and knowledgeable, reliable, trustworthy, timely respond to requests)	Holsapple and Lee-Post (2006); La Rotta et al. (2020); Naveh et al. (2010), student survey
User satisfaction	Positive experiences, recommendation to others, involvement	Holsapple and Lee-Post (2006); McPherson and Nunes (2006)
	Sustainability and up-to-dateness of information, content development & maintenance	Odunaike et al. (2013)
	Platform independence/cross-platform usability (system independence, portals and platforms used by an HEI must be integrated, prevent redundancies and overlaps)	Expert interviews; student survey
Net impact	Learning enhancement & academic achievement	Holsapple and Lee-Post (2006); Raspopovic and Jankulovic (2014)
	Time savings	Holsapple and Lee-Post (2006); Raspopovic and Jankulovic (2014)
	Knowledge gain	Raspopovic and Jankulovic (2014)
	Empowerment	Holsapple and Lee-Post (2006)
	University top management	expert interviews
	Added value (exciting functionalities)	student survey
	Credibility of relevant recommendations (meaningful, well-founded recommendations)	expert interviews
Intention to use	Motivation	Bani-Salameh and Abu Fakher (2015); Mosakhani and Jamporazmey (2010); Selim (2007)
	Usefulness	Hao et al. (2017)
	Willingness to be open to use	Odunaike et al. (2013)
	Social factors (role of peers and lecturers)	Tarhini et al. (2013)
	Self-regulation/organization	Eom and Ashill (2016); Miranda et al. (2014); expert interviews
	Different languages	Lu and Dzikria (2019)
	Defined target groups	expert interviews

## Discussion, implications, recommendations, and a further research agenda

Based on a qualitative and quantitative study and an extensive literature review, we identified 28 CSF and challenges. We structured them using the established IS success model of DeLone and McLean (2016). Our multi-perspective view with various stakeholders and disparate literature allowed us to look at various angles and extract CSF and challenges from various perspectives. The empirical and student-centered approach allowed for openness and impartiality. First allowing students to speak, the rest of the research process evolved into interviewing various experts, such as HEI organizational unit leaders and lecturers, and finally conducting a literature review. We found that CSF and challenges were numerous and diverse. Therefore, we structured them using the established IS success model of DeLone and McLean (2016), which has been tested and iterated for many years, to obtain an appropriate differentiation. In general, the focus of academic research, academic staff, lecturers, and students is highly similar; therefore, many of the identified CSF and challenges are perceived as important by all stakeholders. We have also consistently identified similar CSF and challenges in our literature review, whether we were examining SPA (Knote et al., 2019), e- or m(obile) learning, or web-based learning. The similarity of CSF and challenges across application domains has the advantage that many and various studies can be analyzed, making the result more nuanced, valid, and reliable. It also shows that the specification of the objectives is crucial. Because of the parallels with existing research topics, our results and findings can be used to compare IDSA with other digital assistants, such as SPA or PCA (e.g., Wellhammer et al., 2020).

Students, in particular, demand easy access as they are used to finding a variety of attractive learning apps to help them learn or to share a document with their study group and earn a few euros, such as “studydrive” (https://www.studydrive.net). Students, lecturers, and managers plead for more data protection to receive less advertising. In implementation, this means that users must be encouraged to think about sharing their personal data and act responsibly (cf. self-regulation theory); otherwise, as with other apps, this will result in impulsive decisions (deductive data privacy; Janson et al., 2021). This means that data protection within an IDSA must be designed in such a way that it is clear to users what exactly happens to their personal data; in Europe, this is regulated by the General Data Protection Regulation (GDPR). In addition, reliable quality and timeliness of content builds trust and satisfaction in an IDSA. To improve learning performance, for example, many students use the app Forest to “stay focused, be present” and maintain concentration. Users are asked to plant virtual trees, forcing them to let their smartphone rest for a period of time. Only when the phone is not in use can the tree grow; otherwise, it dies. This promotes concentration and supports academic success saving time.

Some of the identified CSF and challenges cannot be clearly assigned to one dimension only within the IS success model (DeLone & McLean, 2016) because they influence and interact with each other. For example, platform independence/cross-platform usability influences the CSF and challenges, ease of use, ease of access, and time savings. When integrated with a known platform, IDSA is easy to use because the system is already known and no additional registration process is required, saving time in the end. The primary purpose of an IDSA is to improve self-regulation skills, track one’s learning goals, and support study organization providing appropriate functionalities (Carver & Scheier, 2011; Zimmerman, 2012). It is often our experience with students that their ability to study independently without guidance from instructors, to set their own study goals, or to learn a topic on their own is insufficiently developed. Providing these IDSA functionalities, students can use the IDSA to deal with their own goals on a reflective level, with the learning content or more generally with the question “Where do I want to go after the bachelor’s degree?” In addition, reliable and long-term funding for IDSA is fundamental to its selection, adaptation, implementation, operation, maintenance, and evolution and is seen as both a success dimension and a challenge.

Through our research, we contribute to the knowledge base of IDSA and digital assistants in general identifying CSF and challenges that can impact the success of an IDSA. HEI can use our findings and insights to support projects and processes for IDSA selection, adaptation, implementation, operation, maintenance, and improvement. In particular, given the changes in higher education due to the global COVID-19 pandemic, an IDSA has particular potential to provide personalized support to students and make individualized and factual recommendations if it follows certain characteristics, see Table 3. Our results and findings provide insights for IDSA system developers and vendors. The identified CSF and challenges can assist higher education management and faculty in effectively implementing an IDSA. Our multi-perspective study found a high level of agreement among faculty, organizational unit, and student perspectives, although some differences remain. Despite the high level of agreement on many CSF and challenges among all stakeholders, experts and faculty from HEI mainly focus on the critical aspect of support from top leadership, the credibility of relevant recommendations, and self-regulation and organization. Students frequently cited flexibility, qualified staff, quality of responses, responsiveness of staff, no redundant information, and data integration as essential. Therefore, decision makers must consider all stakeholder perspectives when developing an IDSA. As the target audience or users of an IDSA are the students, it is important to consider their needs and involve them in the IDSA development and implementation process. However, for a successful launch, the HEI’s structures must also fit; content for the IDSA must be provided and made available, and faculty must also promote an IDSA. Therefore, a holistic view of the requirements of the various stakeholders is critical for decision makers. The early involvement of all stakeholders also increased the acceptance of the final IDSA. Failure to consider either stakeholder perspective can lead to a lack of acceptance, lack of utilization, and an undesirable IDSA. Fears such as losing one’s job or being made “redundant” are quite realistic.

In addition, our study contributes to IS theory combining knowledge about IDSA with DeLone and McLean’s (2016) IS success model. To further contribute to the knowledge base, we developed a research agenda with nine research directions (Watson & Webster, 2020). We recognized that many of the identified CSF and challenges depend on a HEI’s IT maturity. This determines the complexity of an IDSA’s functionalities; for example, an IDSA with a chatbot requires a higher maturity level than a rule-based one. Further research is needed to further explore these influences and determine what critical processes exist and how they affect the implementation, operation, and use of an IDSA. To this end, further research can develop a maturity model to determine (1) how and what processes of implementing, operating, and using an IDSA are affected by a HEI’s IT maturity level; (2) how an IT maturity model can be structured for the implementation, operation, and use of an IDSA. In addition, we found that data privacy and security for faculty, HEI staff, students, and in the literature is a CSF and challenge for IDSA. However, its implementation faces many challenges. Research is needed in this topic area to define and develop consistent guidelines for privacy-friendly IDSA in HEI; (3) what guidelines can be derived to enable a privacy-friendly and secure IDSA. A study is characterized by different phases of study, all of which can be supported differently by an IDSA (blinded to the review process). Further research is needed to determine for which activities within study phases the need for an IDSA is particularly high and how an IDSA can support them; (4) which activities within a study phase can best be supported by an IDSA and what their critical functionalities are. Due to the heterogeneity of students and study programs, the target group of an IDSA is highly diverse. Information and support needs differ between various target groups of students, e.g., first-year or international students, mechanical engineering students, and student teachers, and future research can systematically analyze these needs and translate them into an IDSA; (5) how the needs of an IDSA differ between various student target groups; (6) what design elements and functionalities an IDSA must provide to support them. In our study, we did not consider IDSA from the field, although these may provide further CSF, challenges, and information on why IDSAs fail or succeed in practice. Further research requires the analysis of IDSA that have already been implemented, and the investigation of IDSA that are no longer in use can also make an important contribution; (7) what CSF can be derived from IDSA in practice; (8) what the reasons for the failure of an IDSA in practice are and what lessons can be learned. Our quantitative and qualitative studies are limited to three German universities. As the structures and conditions at universities vary widely even within Germany, further research needs to analyze and identify possible additional CSF and challenges arising from the differences and content aspects of HEI in different countries and their cultural influences, e.g., data privacy and data security differ from country to country due to various legal requirements and are of interest for cross-cultural analysis; (9) how cultural differences influence the implementation, operation, and use of an IDSA. Table 4 provides an overview of the overall research agenda.

**Table 4:** Further research agenda

Topics for a Further Research Agenda	Research Questions
Many identified CSF and challenges depend on the HEI’s IT maturity level (i.e., to what extent the functionalities can be developed and offered maturely).	(1) How and which processes of an IDSA implementation, operation, and usage are influenced by an HEI’s IT maturity? (2) How can an IT maturity model for an IDSA implementation, operation, and usage be structured?
A significant issue is personal data protection, which is reflected in the literature review and our surveys with all stakeholders. To fulfill this requirement is a major challenge.	(3) What guidelines can be derived to enable privacy-friendly data protection and security for IDSA?
There is a need to clarify in which study phase the need for an IDSA is particularly high and how an IDSA can support it.	(4) Which activities within a study phase can be best supported by an IDSA and what are their critical functionalities?
HEI offer a wide range of study programs such as law, engineering, teaching, economics, and business administration. Future research can systematically analyze these needs and translate them into an IDSA.	(5) How do IDSA requirements differ between various target student groups? (6) What design elements and functionalities must an IDSA provide to support them?
Further research requires to analyze already implemented IDSA. Examining IDSA that are no longer used can also contribute significantly.	(7) What CSF can be derived by real-world IDSA?
It is still necessary to better understand under which conditions an IDSA will fail in practice or when it will be well received and accepted.	(8) What are the reasons for an IDSA to fail in practice and the resulting lessons learned?
Further research must analyze and identify possible more CSF resulting from differences and content-specific aspects of HEI in different countries and their cultural influences.	(9) How do cultural differences influence an IDSA’s implementation, operation, and usage?

## Limitations and conclusions

Despite its scope, our research has some limitations. Our subjective perceptions influenced our literature analysis. We minimized this using inclusion and exclusion criteria and adding a forward-, backward-, author-, and similarity search (Google Scholar). Further, when conducting and analyzing interviews, our results might have been influenced by different or subjective experiences and knowledge. Our research is further limited analyzing German HEI, only; thus, our results and findings are especially applicable to Germany and the transferability to other countries is partially limited. The focus may differ from our identified CSF and challenges in other countries. Moreover, the results of our studies represent only a snapshot and were partially collected before the COVID-19 pandemic. To minimize this influence, we performed the qualitative and quantitative study and literature review from 2019 to 2021. Our identified CSF and challenges result from our qualitative and quantitative studies as well as literature review. However, we did not explicitly evaluate our results and findings, for instance, by a posteriori focus group discussion with HEI experts, lecturers, or students.

Several studies about CSF and challenges for e- and m-learning and web-based learning in literature already exist; however, we found no study that explicitly addresses specific CSF and challenges for an IDSA. Therefore, we deduced various CSF and challenges to support students in strengthening their self-regulation skills, improving their study organization and enabling individualized recommendations with our mixed methods research design. Based on a student survey with 570 participants, 28 HEI expert interviews, and a literature review, we identified 28 CSF and challenges and categorized them within the IS success dimensions of system maturity and quality, information quality, service quality, user satisfaction, net impact, and intention to use proposed by DeLone and McLean (2016).

## Data Availability

The datasets used and/or analysed during the current study are available from the corresponding author on reasonable request.

## Appendix

### Questionnaire quantitative study with students

1. Sociometric data (open text):
  - Course of study
  - Semester
  - Planned degree (Bachelor / Master)
  - Gender
  - Age

2. Which contents should your personal assistant be familiar with so that you experience it as valuable? (multiple answers possible)
  - It knows my subjects.
  - It will tell me about interesting courses, e.g., seminars, lectures, tutorials, projects, labs, outside my home university, e.g., courses offered by other universities.
  - It names study paths that will effectively bring me to my goal, e.g., for the goal I have set myself, to combine meaningful lectures and seminars tutorials, projects, labs, and to suggest learning materials.
  - It names the advantages and disadvantages of my lecture and seminar choices.
  - It names learning groups for me.
  - It accurately names advising offices for specific questions.
  - It provides me with information about a semester abroad.
  - It helps me to organize my study plan.
  - It shares experiences/comments about lectures, seminars, teaching offers, tutorials, projects, labs, etc. of fellow students.
  - It can remind me of assignments, (partial) goals, etc.
  - It makes suggestions about events, e.g., lectures, offered through modern e-learning applications, thus reducing my attendance time at the university.
  - It shall optimize my study plan in terms of content (courses offered) and time (days of attendance at the university).
  - It should also inform me about teaching materials and resources that are freely and openly available, including on the Internet and as openly licensed Open Educational Resources (OER).
  - It shall provide me with exam experiences of fellow students.
  - It informs me about inter-university offers.

3. What attributes should your personal assistant have? (Likert Scale)
  - Gamification elements
  - Language skills
  - Switching between male/female voice
  - Interdisciplinary competence
  - Reminder function
  - Own calendar or link to cell phone calendar and/or LMS timetable
  - Easy to use / user interface self-explanatory
  - Chat or forum function for students
  - OER search engine
  - Free menu design (color scheme, arrangement of individual elements)
  - Humor
  - Factual orientation without gamification elements

4. What else is important to you when you think about this assistant accompanying you personally? (open text)
5. What are the criteria for you not to use a personal assistant? (open text)

### Interview guideline for qualitative study

1. General questions
  - What is your understanding of an individualized digital study assistant (IDSA)?
  - What added values might an IDSA enable in academic education?
  - What risks might occur during an IDSA design and development?

2. Organizational conditions for success
  - What are questions frequently asked regarding study/semester planning?
  - How can an IDSA support students and when would you recommend it?
  - What aspects should be paid special attention to implement an IDSA successfully?
  - (e.g., distribution, sustainability, cooperation, usability, added value)
  - What are organizational framework conditions for an IDSA?
  - In your opinion, are there individual characteristics to which special attention must be paid? (e.g., flexibility, reliability)
  - Where do you see potential barriers to IDSA adoption and usage? (e.g., socio-technological, technological, legally [e.g., data privacy], organizational [internal factors, intra-/inter/extra organizational factors], competence-, and resource-based)
  - Where do you see opportunities to overcome or counteract these barriers?

3. Other question
  - Is there anything else about this topic that is important to you and has not been discussed?
